# Generation of a New Congenic Mouse Strain with Enhanced Chymase Expression in Mast Cells

**DOI:** 10.1371/journal.pone.0084340

**Published:** 2013-12-31

**Authors:** Xi Jin, Wanke Zhao, Kaiyao Shi, Wanting T. Ho, Zhizhuang J. Zhao

**Affiliations:** 1 Department of Pathology, University of Oklahoma Health Sciences Center, Oklahoma City, Oklahoma, United States of America; 2 Department of Cardiology, China-Japan Union Hospital of Jilin University, Changchun, China; Indian Institute of Science, India

## Abstract

Mast cells are effector cells best known for their roles in IgE-associated allergy, but they also play a protective role in defense against pathogens. These cells express high levels of proteases including chymase, tryptase and carboxypeptidase. In the present study, we identified a congenic strain of C57BL/6 mice expressing an extraordinarily high level of chymases Mcp-2 and Mcp-4 in mast cells. The overexpression was associated with variant Mcp-2 and Mcp-4 genes originated from DBA/2 mice that also expressed high levels of the two enzymes. Real time PCR analysis revealed that Mcp-2 and Mcp-4 were selectively overexpressed as tryptases, Cpa3 and several other chymases were kept at normal levels. Reporter gene assays demonstrated that single-nucleotide polymorphisms (SNPs) in the promoter region of Mcp-2 gene may be partly responsible for the increased gene transcription. Our study provides a new model system to study the function of mast cell chymases. The data also suggest that expression of chymases differs considerably in different strains of mice and the increased chymase activity may be responsible for some unique phenotypes observed in DBA/2 mice.

## Introduction

Mast cells are innate immune cells best known for their involvement in anaphylaxis, atopic asthma and other IgE-associated allergic disorders [1]. They also carry out a number of beneficial functions to the host including immune responses toward various pathogens. They are derived from hematopoietic stem cells and are widely distributed in tissues. Mast cells express a number of proteases including chymase, tryptase, and carboxypeptidaseA [2]. In mice, Mcp-1, -2, -4, -5, -9, and -10 are designated as chymases based on deduced amino acid sequences, whereas Mcp-6 and -7 are tryptases. These enzymes are stored in high amounts as active enzymes in mast cell secretory granules. Upon activation, massive fully active mast cell proteases are released through mast cell degranulation and elicit essential impacts on many physiological and pathological events which include extracellular matrix remodeling, extravascular coagulation, fibrinolysis, angiogenesis as well as antibacterial inflammatory responses [3].

Expressions of chymases are strictly regulated. At the level of transcriptional regulation, a well-documented transcription factor is Mitf. Direct or indirect binding of Mitf to the promoter element CANNTG can significantly enhance the expression of Mcp-2, -4, -5, -6, and -9 genes in C57BL/6 mice [4]. In addition to Mitf, bifunctional transcription factors C/EBPβ and YY1 are thought to be responsible for the negative transcriptional regulation of Mcp-2 via intracellularly retained IL-15 [5], [6]. In wild type bone marrow-derived mast cells (BMMCs), C/EBPβ is preferentially expressed over YY1 and binds to the Mcp-2 promoter. In contrast, in IL-15-deficient BMMCs, YY1 is dominantly expressed and binds to the Mcp-2 promoter, which allows hyper-transcription of the Mcp-2 gene [5]. Expression of chymases in mast cells is also known to be controlled at the post-transcriptional level. For example, an earlier study demonstrated that the half-life of the Mcp-2 transcript in mouse BMMCs was extended by 4-fold in the presence of IL-10 [7]. Together, expressions of chymases are regulated at multiple levels.

We previously generated a line of JAK2V617F transgenic mice that display polycythemia vera-like phenotypes [8]. Our most recent work demonstrated that the occurrence of PV-associated pruritus in these mice was associated with elevated levels of mast cells (Jin et al, unpublished). In this study, we identified a subpopulation of JAK2V617F transgenic mice that express very high levels of Mcp-2 and Mcp-4 in mast cells. However, this was found to be independent of JAK2V617F and due instead to the presence of Mcp-2 and Mcp-4 gene variants originated from DBA/2 mice. Our study thus provides a new line of congenic C57BL/6 mice with high expressions of specific chymases in mast cells.

## Materials and Methods

### Mice

JAK2V617F transgenic mice were generated with a C57BL/6×DBA/2 hybrid background and then crossed with wild type C57BL/6 mice for over 10 generations [8]. Wild-type C57BL/6 and DBA/2 mice were purchased from The Jackson Laboratory. Animals were housed in ventilated cages under standard conditions. This study was carried out in strict accordance with the recommendations in the Guide for the Care and Use of Laboratory Animals of the National Institutes of Health. The protocol was approved by the Institutional Animal Care and Use Committee of the University of Oklahoma Health Sciences Center.

### Culture of Mast Cells

Bone marrow and peritoneal cavity cells from mice were cultured in Iscove’s modified Dulbecco’s medium (IMDM) supplemented with 20% fetal bovine serum (FBS) and 1% each of conditioned media of cultured CHO cells overexpressing mIL-3 and mSCF. The resultant mast cells were analyzed after one month of culture initiation and maintained for up to four months with equal volumes of fresh medium added every 3 to 5 days. These cells were >95% pure based on positive staining for CD117 (c-Kit) and FcεR1 upon flow cytometric analyses.

### Proteomic Analyses

Protein identification was carried out by using the Mass Spectrometry and Proteomics core facility at the University of Oklahoma Health Sciences Center. In brief, proteins were separated on SDS gels, and protein bands were excised for digestion with trypsin. This was followed by HPLC separation with a Dionex UltiMate 3000 LC system and MS/MS analysis with an ABI MDS Sciex Qstar Elite mass spectrometer. MS/MS data was collected with the ABI Analyst QS 2.0 software and analyzed by using the Mascot search engine (Matrix Science) for protein identification against the 2011 SwissProt protein database.

### Isolation of DNA and RNA

Genomic DNAs were purified from cultured mast cells and mouse tails by using the phenol/chloroform extraction method following digestion of samples with proteinase K. Total RNAs were isolated from cultured BMMCs by using the RNeasy Mini Kit (Qiagen), and single strand cDNAs were synthesized by using the QuantiTect reverse transcription kit from Qiagen.

### Protein Expression and Antibody Production

DNA fragments encoding the mature Mcp-2 and Mcp-4 proteins without N-terminal signal sequences were amplified from mast cell single-strand cDNA by PCR with primer sets 5′-gaggagattattggtggtgttgagg plus 5′-ggcttttcagctacttgctctttaa and 5′-gaggagattattggtggtgttgagt plus 5′-ggcttttcactacttgccctttata, respectively. The PCR products were cloned into the pBluescript KS vector, and inserts were verified by DNA sequencing. This was followed by subcloning of the DNA inserts into a pT7 vector for protein expression as non-fusion proteins. Protein expression in recombinant *E. coli* cells was induced by 1 mM isopropyl β- D -1-thiogalactopyranoside (IPTG). Both Mcp-2 and Mcp-4 proteins were found as prominent proteins in the inclusion body of the cells. We thus employed preparative SDS gels to purify them to near homogeneity. The purified proteins were used to immunize mice for generation of mouse anti-sera which were directly used for subsequent western blotting and immnuofluorescent cell staining.

### PCR and Molecular Cloning

The entire coding cDNA sequence of Mcp-2 and Mcp-4 were amplified from mast cell single-strand cDNA. The primers were 5′-ggcaaaatgcaggccctactatt and 5′-gggatgaactcagaggtaccagatg for Mcp-2, and 5′-ggcaagatgcaggccctactatt and 5′-gactctgatgcacgcaggtcagg for Mcp-4. The PCR products were purified and sequenced from both directions. DNA fragments flanking the 5′ coding sequences of Mcp-2 and Mcp-4 genes, designated Mcp-2P and Mcp-4P, were amplified from mouse genomic DNAs by using primers sets 5′-gaagctgctctcaaccttgcgtcag plus 5′-tttgccagtgttgaggccttggtg and 5′-cttgccagtgtcggtcacagcttg plus 5′-gctgttctcaacctatagatcacaacc, respectively. Allele-specific PCR was used to detect nucleotide variations or SNPs in the Mcp-2P region. The primers used are 5′-ctcacactggtcaacacaaacatta and 5′-tctgctgttaaacacaaacacagtct for the normal form, and 5′-ctcacactggtcaacacaaacattg and 5′-tctgctgttaaacacaaacacagtca for the variant form. Restriction fragment length polymorphism (RFLP) was employed to detect a nucleotide substitution in the Mcp-4P region. For this purpose, the Mcp-4P PCR product was digested with restriction enzyme NdeI. The variant form of Mcp-4P gave rise to two fragments while the normal form of Mcp-4P was not cleaved. For report gene assays, Mcp-2P was cloned into the pGL3 luciferase reporter vector. For cloning of Mitf-A, a DNA fragment encoding the full-length form of Mitf-A was amplified by PCR using primers 5′-ggagtcatgcagtccgaatcgg and 5′-tcctgaagaagagagggagcggt with mast cell single strand cDNAs as template. The PCR products were cloned into the pBluescript KS vector, sequence-verified, and then subcloned into the pcDNA3 vector for expression in mammalian cells under the CMV promoter.

### SDS-PAGE and Western Blot Analyses

Cultured mast cells were collected and lysed in a buffer containing 25 mM β-glycerophosphate (pH 7.3), 5 mM EDTA, 2 mM EGTA, 5 mM β-mercaptoethanol, 1% Triton X-100, 0.1 M NaCl, and a protease inhibitor mixture or in 1X SDS sample buffer. Proteins were resolved on 10% or 12.5% SDS gels and then stained with Coomassie blue R-250 or transferred to polyvinylidenedifluoride (PVDF) for western blotting with anti-Mcp-2 and anti-Mcp-4 antibodies followed by horseradish peroxidase-conjugated secondary antibodies. Enhanced chemiluminescence signals were captured by using the FluorChem SP imaging system from Alpha Innotech.

### Immunofluorescent Cell Staining

Cultured mast cells were spun onto glass slides by cytocentrifugation and fixed with 4% formaldehyde in PBS for 20 minutes. For antigen retrieval, fixed cells were treated with a buffer containing 10 mM sodium citrate (pH 6.0) and 0.05% Tween 20 for 40 minutes at 95–100°C. After rinsing with PBS, cells were probed with primary anti-Mcp-2 and Mcp-4 antibodies for 2 hours and then with a Cy3-conjugated anti-mouse secondary antibody for 1 hour. The nucleus was stained with 0.1 µg/ml Hoechst 33258. Fluorescence was visualized under 40X or 100X lens with an Olympus BX51 fluorescent microscope. Images were captured by using a DP71 digital camera.

### Chymase Activity Assays

Cultured mast cells were collected and washed with ice-cold PBS. Following lysis in a buffer containing 25 mM Tris-HCl (pH 8.5), 1% Triton X-100, 5 mM EDTA and 0.1 M NaCl, cell extracts were cleared of insoluble materials by centrifugation at 18,000×g for 10 min. Chymase assays were performed with 0.375 mg/ml substrate N-Succinyl-Ala-Ala-Pro-Phe p-nitroanilide (Sigma-Aldrich) in 0.1 M Tris-HCl (pH 8.0). The reaction was allowed to proceed at room temperature, and absorbance was read at 405 nm using a nanodrop spectrophotometer at various time points. To calculate enzymatic activity, molar extinction coefficient 9.5×10^3^/M/cm was used.

### Degranulation of Mast Cells

Degranulation of mast cells was achieved by ligation of the high-affinity IgE receptor FcεR1 via IgE. For this purpose, cultured mast cells were sensitized with 0.15 µg/ml of anti-DNP IgE (Sigma-Aldrich) in complete culture medium overnight at 37°C. Cells were then washed twice with and re-suspended in plain IMDM medium. This was followed by stimulation with 0.05 µg/ml DNP-HSA for 30 min at 37°C. Cell and medium were then separated. Chymase activity in the medium and that remained in cells were determined as described above.

### Real Time PCR Analyses

Total RNAs were isolated from cultured BMMCs by using RNeasy Mini Kit (Qiagen), and 1 µg RNA was then used to synthesize single-strand cDNA by using the QuantiTect reverse transcription kit from Qiagen. Real time PCR was performed in an IQ5 Multicolor Real-Time PCR Detection System using iQ SYBR Green Supermix (Bio-Rad). PCR amplifications were performed in triplicates, and the conditions were 95°C 20″, 59°C 20″, and 72°C 20″ for 45 cycles. Melting curves were analyzed to confirm specific amplification of desired PCR, and the identities of final PCR products were verified by separation on agarose gels and by DNA sequencing. For quantification, standard curves were obtained by performing PCR with serial dilutions (covering 5 orders of magnitudes) of purified PCR products in salmon sperm DNA. Levels of transcripts were normalized against that of mouse glyceraldehyde-3-phosphate dehydrogenase (GAPDH).

### Cell Transfection and Report Gene Assays

For reporter gene assays, pGL3 luciferase reporter constructs together with the pRL-TK Renilla luciferase control vector were used to transfect mast cells and NIH3T3 cells. Transfection of mast cells was carried out by using the BTX Systems 600 Electro Cell Manipulator with a single 8.4 ms pulse with the setting of 800 µF, 350 V, and R1–13. Transfection of NIH3T3 cells was performed by using the Fugene 6 transfection reagent (Roche Applied Science). NIH3T3 cells were co-transfected with pcDNA or pcDNA3-Mitf-A. Luciferase activity was measured 24 hr after cell transfection by using the Dual-luciferase Reporter Assay System (Promega). Firefly luciferase activity was normalized against renilla luciferase activity.

### Statistical Analysis

Statistical analyses were performed using the GraphPad Software. Differences between 2 groups of samples were assessed using t tests. p values less than 0.05 (2-tailed) are considered significant.

## Results

### Identification of Markedly Increased Expressions of Mcp-2 and Mcp-4 in Mast Cells Derived from a Subpopulation of JAK2V617F Transgenic Mice

**Figure 1:**
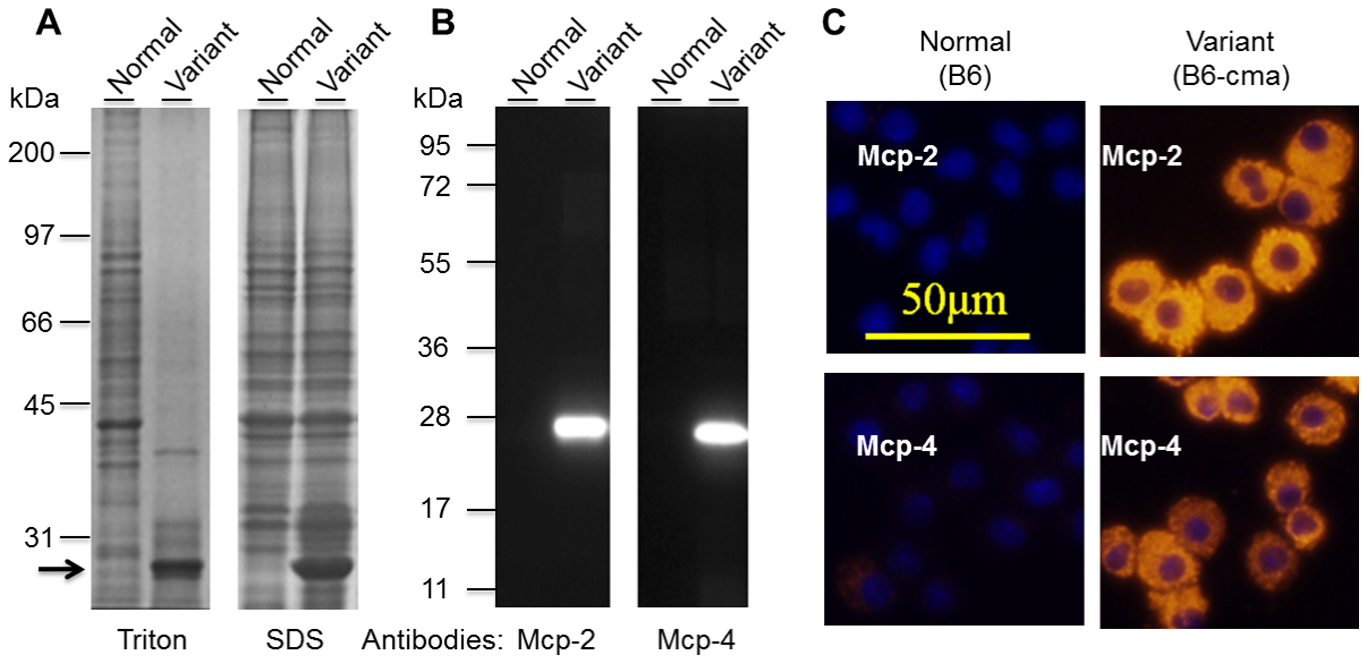
Identification of markedly increased expressions of Mcp-2 and Mcp-4 in BMMCs from a subpopulation of JAK2V617F transgenic mice. **A.** Detection of a predominant protein band in cell extracts of BMMCs from a variant line of mice. BMMCs from two JAK2V617F transgenic mice were extracted in a buffer containing 1% Triton X-100 or 1X SDS gel sample buffer were resolved on 10% SDS gel, and proteins were visualized by Coomassie blue staining. The arrow points to a predominant band. **B.** Verification of Mcp-2 and Mcp-4 over-expressions by Western blotting with specific antibodies. Extracts of BMMCs were separated on 12.5% SDS gel and subjected to Western blotting analyses with anti-Mcp-2 and Mcp-4. **C.** Verification of Mcp-2 and Mcp-4 over-expression by immunofluorescent cell staining. Mcp-2 and Mcp-4 were probed with specific antibodies followed by Cy-3-conjugated secondary antibodies (red). The nuclei (blue) were revealed by staining with Hoechst 33258.

In a previous study, we generated a line of JAK2V617F transgenic mice that displayed phenotypes resembling polycythemia vera in humans [8]. The mice had an initial C57BL/6×DBA/2 hybrid background but have been crossed with wild type C57BL/6 mice for over 10 generations. Theoretically, they have at least 99.95% C57BL/6 background. Our subsequent studies demonstrated that these mice developed pruritus associated with increased numbers of mast cells (Jin et al, unpublished). Interestingly, during our analyses of proteins extracted from cultured mast cells, we observed a very peculiar phenomenon. In Triton X-100 extracts of BMMCs from a subpopulation of transgenic mice, severe protein degradation occurred after a short incubation of cell extracts at room temperature even in the presence of protease inhibitors. Subsequently, only one major protein band with molecular size of 27 kDa was seen on SDS gels (Fig. 1A, left panel). When cells were extracted in the SDS gel sample buffer, protein degradations were eliminated but the 27 kDa band remained prominent, representing about 20% of total cellular proteins (Fig. 1A, right panel). The data suggest the presence of highly expressed proteins with possible protease activities. To identify the strongly expressed protein or proteins, in-gel trypsin digestion was conducted. This was followed by HPLC separation and MS/MS analyses. Searching of MS data against protein databases by using the Mascot search engine revealed mast cell protease Mcp-2 as by far the best hit with a score of 1612 in comparison with the second best hit actin with a score of 252. Among the top hits was also mast cell protease Mcp-4 with a score of 81. Mcp-2 and Mcp-4 both have expected molecular sizes of about 27 kDa and are most relevant to mast cells. Therefore, they were chosen for further verification.

To determine the expression of Mcp-2 and Mcp-4 in mast cells, we generated polyclonal antibodies by immunizing mice with proteins purified from recombinant *E. coli* cells. The antibodies were highly specific with essentially no cross-reactivity toward each other’s antigen although Mcp-2 and Mcp-4 share 66% sequence identity (data not shown). As expected, western blotting analyses with the antibodies revealed extremely high levels of Mcp-2 and Mcp-4 expression in BMMCs from the variant population of transgenic mice but hardly anything in those from normal mice. Note that the antibody-recognized protein bands had expected molecular sizes with Mcp-4 running slightly below Mcp-2 on SDS gels (Fig. 1B). Immunofluorescent cell staining further confirmed the uniform overexpression of these enzymes in cultured mast cells from this subpopulation of mice and also revealed the apparent distributions of the expressed enzymes in the granules of cells (Fig. 1C). Additional experiments demonstrated that Mcp-2 and Mcp-4 were only overexpressed in mast cells but not in other cells from bone marrow or peripheral blood (data not shown). Together, we identified a variant line of mice that express very high levels of chymases Mcp-2 and Mcp-4 in mast cells. We designate this line of mice B6-cma in reference to their genetic background C57BL/6, or B6 for short.

### Enhanced Expression of Mcp-2 and Mcp-4 is Associated with their Gene Variants

**Figure 2:**
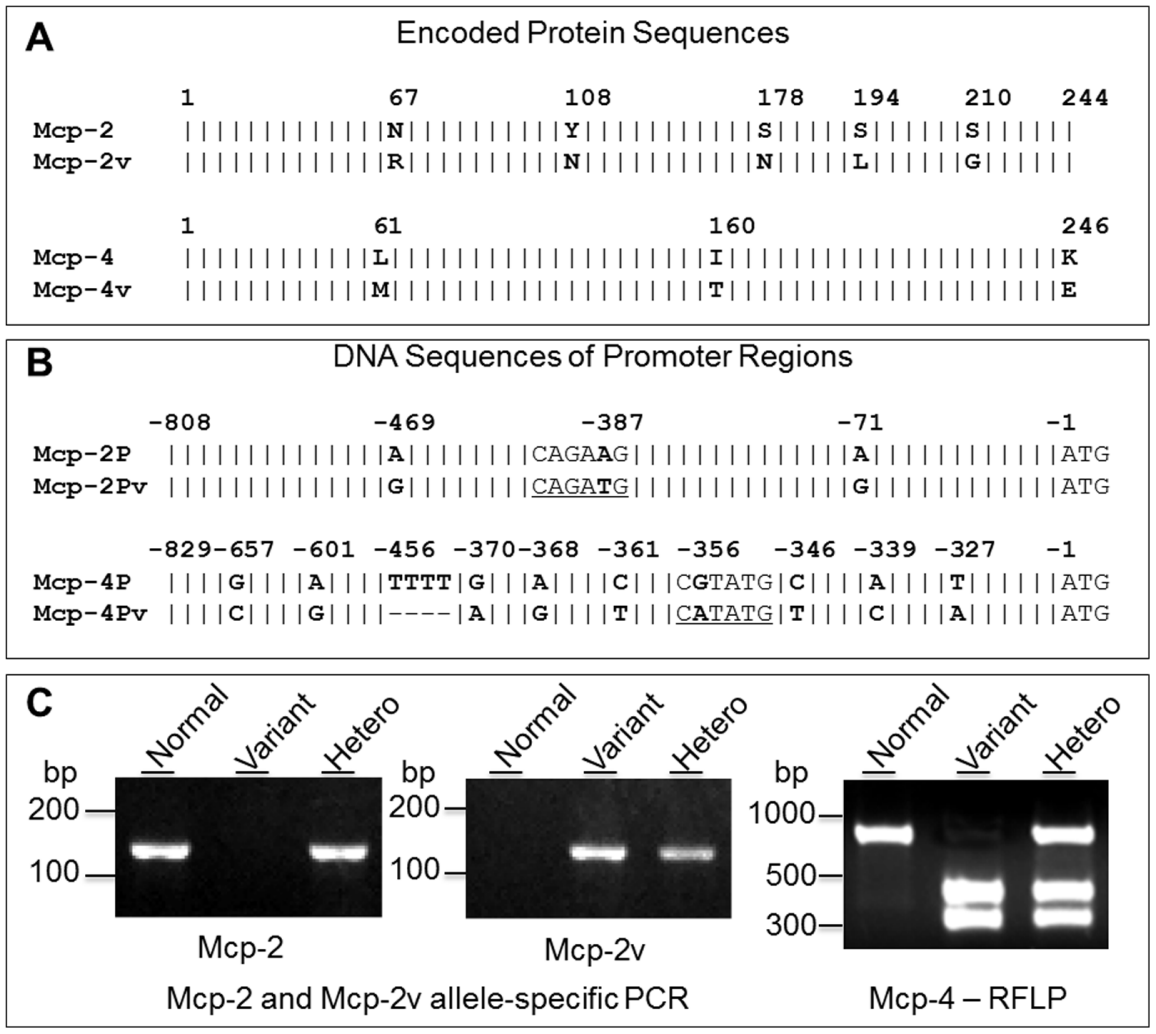
Identification of Mcp-2 and Mcp-4 gene variants. Schematic alignment of amino acid sequences (A.) and promoter region DNA sequences (B.) of Mcp-2P and Mcp-4P from control B6 and variant B6-cma mice. Variant amino acid residues and nucleotide bases are highlighted in bold. A vertical line “|” denotes identical amino acids or bases, and a dash “–” stands for deletions. Putative Mitf binding consensus motifs (CANNTG E-boxes) and an NdeI restriction cleavage site (CATATG) in the variant form of Mcp-2 or Mcp-4 are underlined. **C.** Detection of Mcp-2 and Mcp-4 gene variations in the promoter regions by allele-specific PCR and NdeI restriction fragment length polymorphism (RFLP), respectively. Normal form of Mcp-2P was detected by PCR with primers 5′-ctcacactggtcaacacaaacatta and 5′-tctgctgttaaacacaaacacagtct, while the Mcp-2P variant was amplified by using primers 5′-ctcacactggtcaacacaaacattg and 5′-tctgctgttaaacacaaacacagtca. The expected product size for both is 131 bp. The variant form of Mcp-4P was revealed by NdeI digestion which gave rise to two fragments while the normal form of Mcp-4P was not cleaved. Data show results for both homozygous and heterozygous mice.

Since elevated expression of Mcp-2 and Mcp-4 was found in mast cells from only a subpopulation of JAK2V617F transgenic mice, we thought that it may not be caused by JAK2V617F but rather by other gene alterations gained during the generation of transgenic mice. Interestingly, in the cloning of Mcp-2 and Mcp-4 cDNAs for protein expression and antibody production, we noticed variations in coding sequences of these two enzymes from different mast cells. This likely represents existence of gene variants. To verify this, we amplified cDNAs encoding the full-length forms of Mcp-2 and Mcp-4 from mast cells with or without overexpression of these two enzymes and compare their sequences with the GenBank database. Sequencing analyses revealed that between these two lines of mice, Mcp-2 and Mcp-4 coding sequences differed both by 8 bases, corresponding to 5 and 3 amino acid substitutions in encoded protein sequences, respectively (Fig. 2A). The sequences from normal B6 mice matched entirely the GenBank inputs with access numbers of NM_008571 and NM_010779.2 for Mcp-2 and Mcp-4, respectively. In contrast, sequences from the B6-cma line mice with high level expressions of Mcp-2 and Mcp-4 matched database sequences with access numbers of J05177.1 and M55617.1, respectively, which both correspond to sequences of cDNAs isolated from Kirsten sarcoma virus-immortalized mouse mast cells likely derived from DBA/2 mice [9]–[11].

We further amplified DNA sequences flanking the 5′ end of Mcp-2 and Mcp-4 coding sequences by performing PCR with genomic DNAs as templates. The amplified regions were designated Mcp-2P and Mcp-4P. Their DNA sequences cover putative promoter regions of correspondent genes based on sequencing alignment of multiple mouse chymase genes, transcription prediction programs (e.g., TRANSFAC ver. 7.0), and published literatures [4], [5], [12]–[14]. Counting from the translation start site, Mcp-2P covers −1 to −808 of the Mcp-2 gene with the putative transcription initiation site at −31 and the conserved TATA box at −63, while Mcp-4P covers −1 to −829 of the Mcp-4 gene with the putative transcription initial site located at position −36 and the conserved TATA box at −68. DNA sequencing revealed 3 single-base substitutions in Mcp-2P and 9 single-base substitutions and a consecutive 4-base deletion in Mcp-4P (Fig. 2B). These nucleotide variations or SNPs may be responsible for the enhanced expressions of these enzymes in B6-cma cells. They also provided markers for us to identify the variant genes. Indeed, by performing PCR with allele-specific primers, we were able to identify the normal and variant forms of Mcp-2, and restriction fragment length polymorphism (RFLP) enabled us to distinguish normal Mcp-4 from its variant since a single base substitution happens to create a NdeI site in the variant form of Mcp-4 (Fig. 2C).

**Figure 3:**
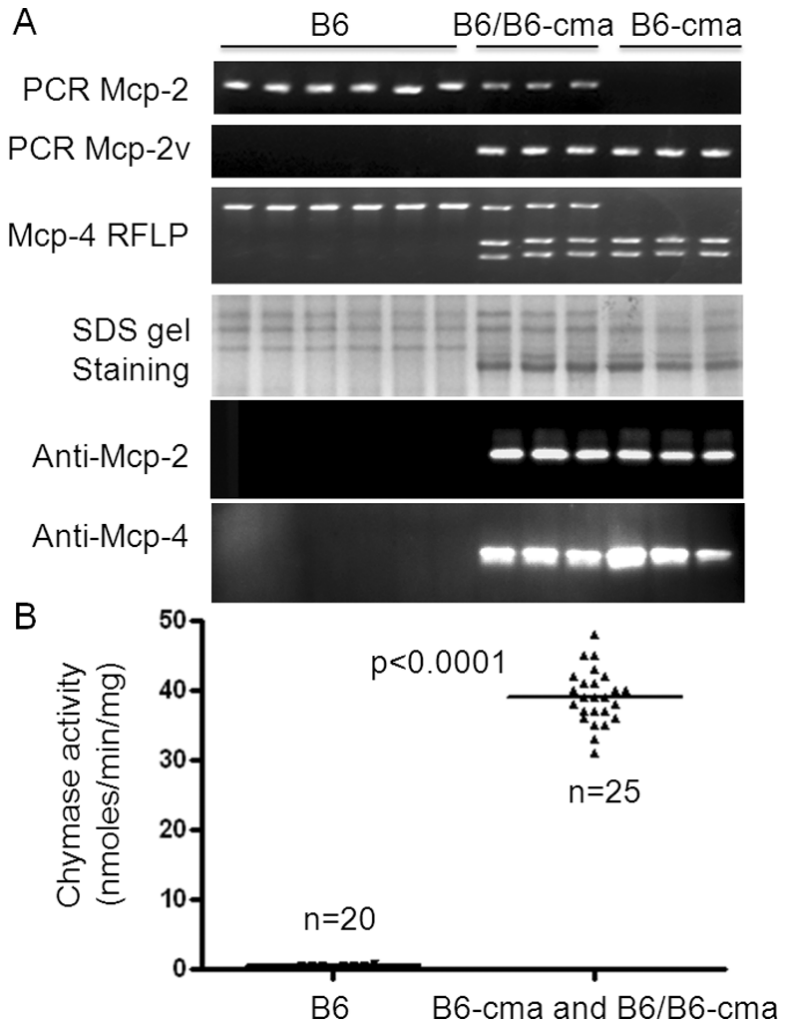
Association of Mcp-2 and Mcp-4 overexpressions in BMMCs with gene variants. **A.** Mice were genotyped for the Mcp-2 gene variant by allele-specific PCR and the Mcp-4 gene variant by restriction fragment length polymorphism (RFLP) with NdeI. BMMCs derived from these mice were analyzed for Mcp-2 and Mcp-4 protein expressions by Commassie blue staining and western blotting. **B.** BMMCs from B6 and B6-cma mice were analyzed for chymase activity. In at least 45 mice analyzed, there is a perfect correlation of Mcp-2 and Mcp-4 gene variants with overexpression of Mcp-2 and Mcp-4 and increased chymase activity.

We further employed the techniques for detection of Mcp-2 and Mcp-4 gene variants to track the lineage of mice produced after crossing of wild type C57BL/6 mice with the subpopulation of JAK2V617F transgenic mice with high expression of Mcp-2 and Mcp-4. We thereby obtained mice carrying variant Mcp-2 and Mcp-4 genes without JAK2V617F. Analyses of protein expression in cultured BMMCs revealed a perfect correlation of the Mcp-2 and Mcp-4 variants with overexpresison of the Mcp-2 and Mcp-4 proteins in over 60 mice with about equal representations of genotypes. Fig. 3A shows representative data obtained from six B6 control and six B6-cma mice. The overexpression of Mcp-2 and Mcp-4 was seen in mice carrying both homozygous and heterozygous copies of the variant genes, suggesting the dominant expression of these variant genes. Furthermore, the overexpression of Mcp-2 and Mcp-4 was also accompanied by significantly increased total chyamse activity in extracts of BMMCs (Fig. 3B). On average, chymase activity in B6-cma mice increased over 40-fold. We thus generated a C57BL/6 congenic mouse line with markedly enhanced expression of Mcp-2 and Mcp-4 in mast cells, which is associated with the genotype of Mcp-2 and Mcp-4 and is independent of JAK2V617F.

### Mcp-2 and Mcp-4 Gene Variants in B6-cma Mice are Originated from DBA/2 Mice

**Figure 4:**
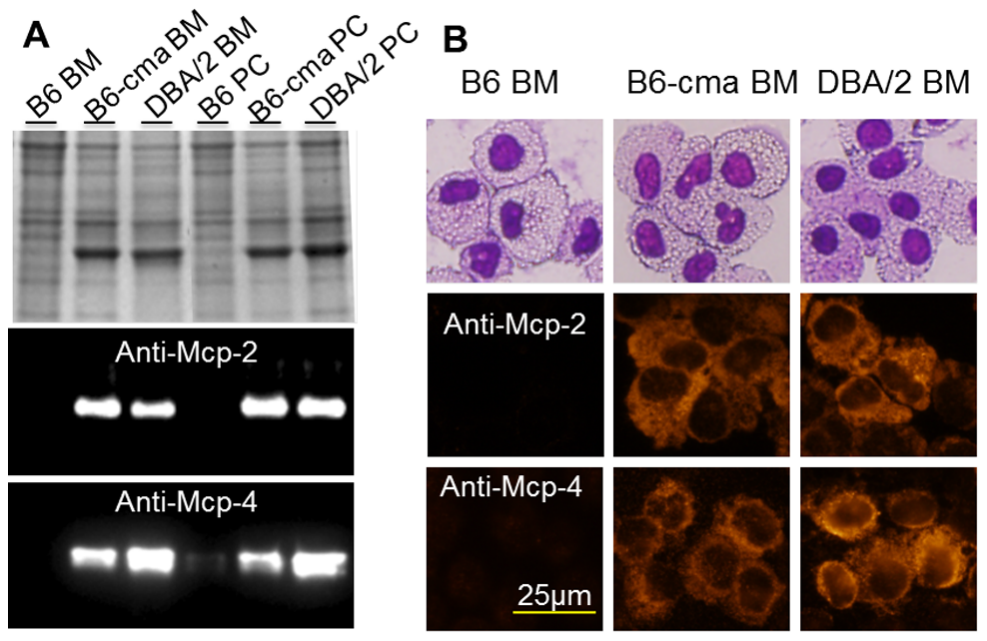
Comparison of Mcp-2 and Mcp-4 protein levels in mast cells from B6, B6-cma, and DBA/2 mice. Mast cells were derived from bone marrow (BM) and peritoneal cavity (PC) of B6, B6-cma, and DBA/2 mice. **A.** Cell extracts were resolved on 12.5% SDS gels followed by Coomassie blue staining (top panel) or western blotting with indicated antibodies. **B.** Cells were subjected to Wright-Giemsa staining (top panel) or immunofluorescent staining with indicated antibodies.

**Figure 5:**
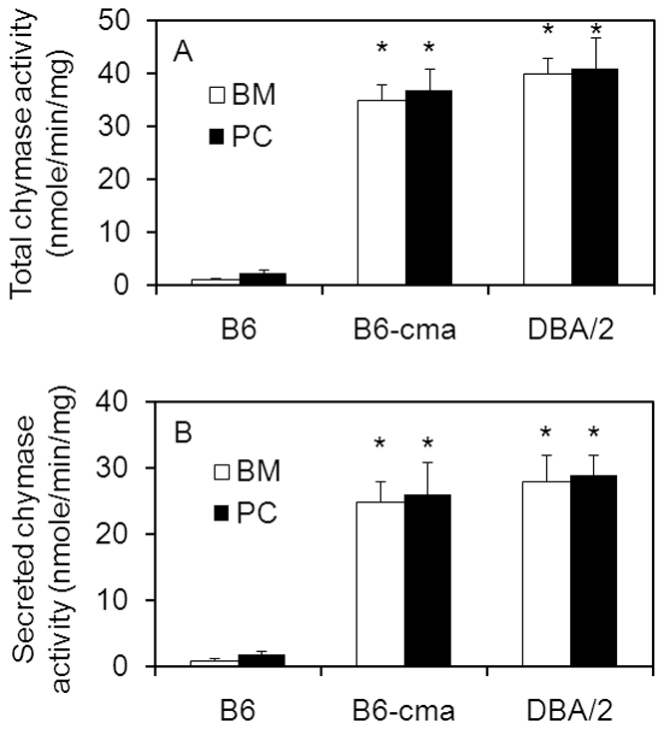
Chymase activity in mast cells from B6, B6-cma, and DBA/2 mice. Mast cells were derived from bone marrow (BM) and peritoneal cavity (PC) of B6, B6-cma, and DBA/2 mice. Cells were either extracted for assays of total chymase activity (A) or treated with antigen to induce degranulation for determination of secreted chymase activity (B). Specific activity was calculated in reference to total proteins in cell pellets. Error bars denote standard deviation (n≥3). *p<0.0001 in comparison with B6 control mice.

Our JAK2V617F mice were initially generated with a C57BL/6×DBA/2 hybrid background. Knowing that C57BL/6 mice carry normal Mcp-2 and Mcp-4 genotypes, we wonder if the variant genes found in our B6-cma mice are originated from DBA/2 mice. We first analyzed the Mcp-2 and Mcp-4 genotypes of DBA/2 mice. DNA sequencing revealed that the coding sequences and 5′ flanking promoter regions of Mcp-2 and Mcp-4 from DBA/2 mice perfectly matched those from our B6-cma mice, indicating that these variant genes indeed originated from DBA/2 mice. We further analyzed the expression of protein and chymase activity in mast cells from these mice. Protein staining, western blotting, and immunofluorescent cell staining revealed that BMMCs from DBA/2 mice showed essentially the same level of Mcp-2 and Mcp-4 overexpression as seen in B6-cma mice (Fig. 4). For comparison, we also analyzed mast cells derived from peritoneal cavity of mice and obtained similar results. Despite the strikingly different levels of Mcp-2 and Mcp-4 expressions, cultured mast cells derived from B6-cma and DBA/2 mice displayed morphologies highly similar to those obtained from control B6 mice (Fig. 4B, top panel). Chymase activity assays also showed expected results with DBA/2 and B6-cma mast cells showing much elevated activity over the control B6 mice cells (Fig. 5A). Mast cell proteases are known to be secreted upon stimulation. To verify the functionality of these overexpressed Mcp-2 and Mcp-4, we induced degranulation of mast cells derived from bone marrow and peritoneal cavity by treating anti-DNP IgE-charged mast cells with the DNP-HSA antigen. This caused over 70% release of total chymase activity into the medium and resulted in essentially proportional levels of secreted chymase in the medium (Fig. 5B). Western blotting analyses also demonstrated significantly higher levels of Mcp-2 and Mcp-4 proteins in the medium (data not shown). This suggested that overexpressed Mcp-2 and Mcp-4 in B6-cma and DBA/2 mice are fully functional. Together, our data demonstrate that the variant Mcp-2 and Mcp-4 genes associated with overexpression of these enzymes in our B6-cma mice originated from DBA/2 mice.

### Transcripts of Mcp-2 and Mcp-4 are Selectively Up-regulated in BMMCs from B6-cma and DBA/2 Mice

**Figure 6:**
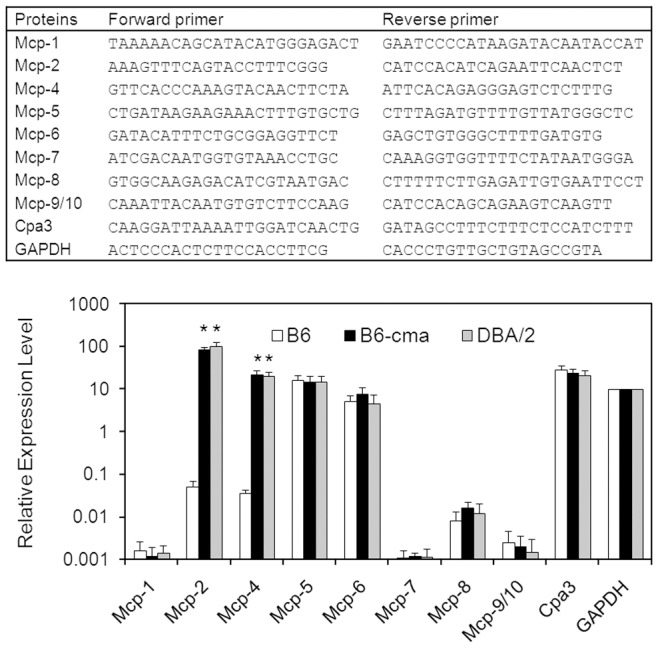
Expression of mast cell proteases in mast cells derived from B6, B6-cma, and DBA/2 mice. Expression of indicated mast cell proteases together with house-keeping gene GAPDH (glyceraldehyde 3-phosphate dehydrogenase) was analyzed by real time PCR using specific PCR primers shown in the top panel. Data represent relative gene expression levels calculated based on threshold cycles and standard curves obtained with serial dilutions of purified PCR products. Error bars denote standard deviation (n≥3). *p<0.0001 in comparison with B6 control mice.

We thought that the increased protein expression of Mcp-2 and Mcp-4 may be caused by elevated levels of mRNA. We performed real time PCR to analyze the transcripts of Mcp-2 and Mcp-4 in cultured mast cells. Together, we analyzed a total of 9 mast proteases together with GAPDH as a control. Among these proteases, Mcp-1, 2, 4, 5, 8, 9, and 10 are clustered on chromosome 14, and they all share sequence similarity with the human chymase, although Mcp-5 was shown to have only elastase-like activity [2]. Mcp-6 and 7 are tryptases, and Cpa represents mast cell carboxypeptidase A and has exopeptidase activity. Since Mcp-9 and Mcp-10 are highly similar in DNA sequences, they were analyzed by using shared primers. Furthermore, in primers for amplification of Mcp-2 and Mcp-4, the aforementioned SNPs were avoided. The sequences of PCR primers are shown in Fig. 6 (top panel). The amplified PCR products were about 80 bp in length. They displayed single peaks upon melting cure analyses and showed single bands on agarose gels. Their identities were further verified by DNA sequencing. Real-time PCR data revealed that transcript levels of Mcp-2 and Mcp-4 were selectively increased by over 1,000-fold in B6-cma and DBA/2 mice in comparison with the B6 control (Fig. 6, bottom panel). The expression levels of all other proteases were comparable in mast cells from B6, B6-cma, and DBA/2 mice. Note that the transcripts of Mcp-5, Mcp-6, and Cpa were expressed at high levels in these mice, while those of Mcp-1, Mcp-7, and Mcp-9/10 were lower. The low expression observed was unlikely caused by poor primer selection because amplification with alternate sets of primers gave similar results (data not shown). Together, the data indicated that Mcp-2 and Mcp-4 are selectively overexpressed in mast cells from B6-cma and DBA/2 mice.

### Reporter Gene Assays Reveal a Potential Role of SNPs in the Promoter Region of Mcp-2

**Figure 7:**
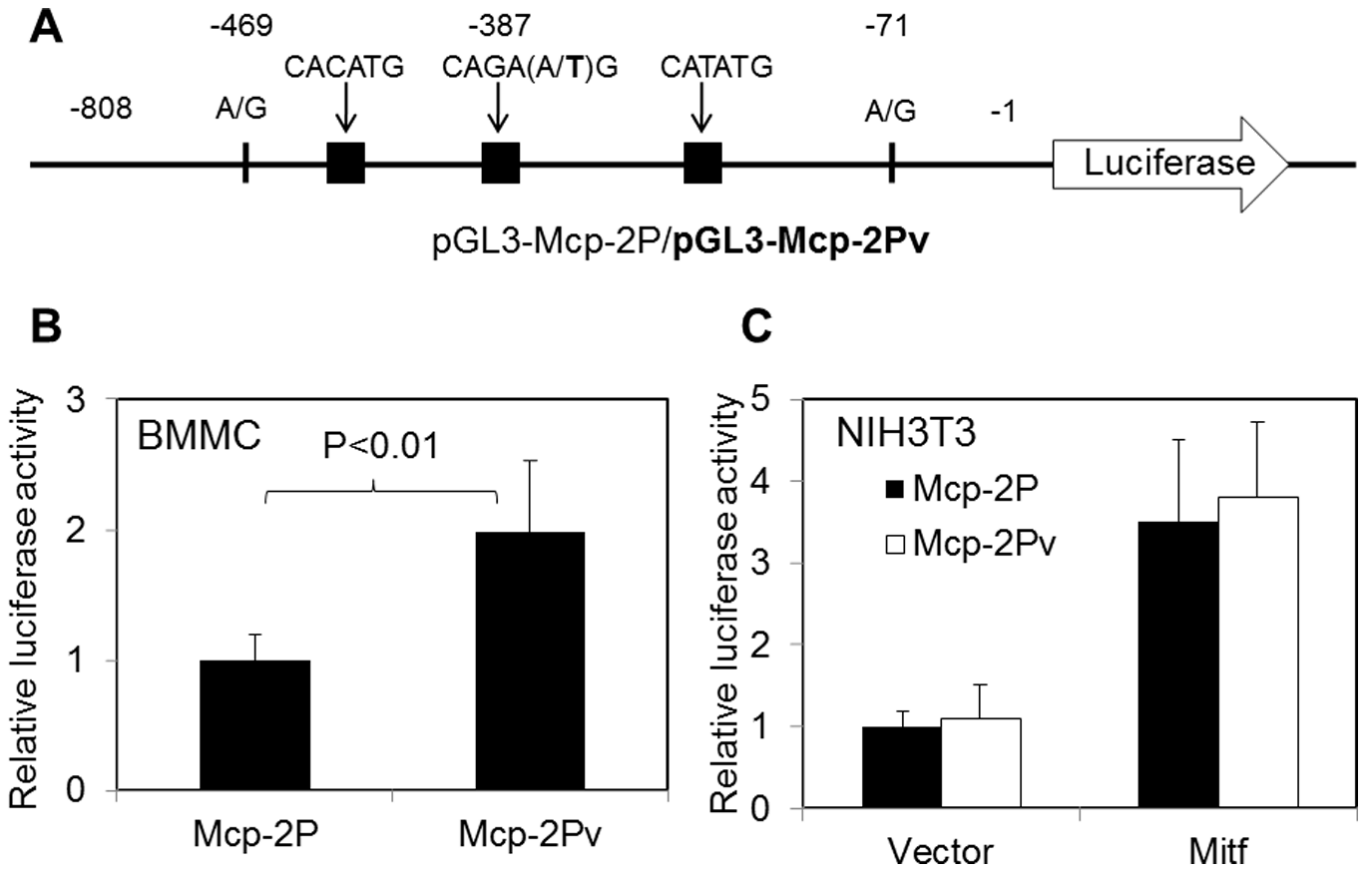
Reporter gene assays. **A.** Schematic diagram of report gene constructs. SNPs are indicated, and black blocks represent CANNTG motifs (E-boxes). **B.** Report gene constructs were transfected into mast cells derived from bone marrow of B6 mice. **C.** Report gene constructs together with pcDNA3 plain vector or pcDNA3-Mitf-A were transfected into NIH3T3 cells. Relative report gene expression is represented by firefly luciferase activity normalized against that of renilla luciferase. Error bars denote standard deviation (n≥3).

The presence of SNPs in the promoter regions of Mcp-2 and Mcp-4 genes suggests a potential alteration of promoter activity. To evaluate if this is responsible for the overexpression of Mcp-2 and Mcp-4 in mast cells, we built reporter gene constructs containing Mcp-2P and Mcp-2Pv, DNA fragments corresponding to the putative promoter region of normal and variant forms of Mcp-2, respectively (Fig. 7A). When introduced into cultured BMMCs by electroporation, the Mcp-2Pv plasmid produced a significantly higher luciferase activity than the Mcp-2P plasmid, suggesting that the 3 SNPs in the promoter region contribute to the enhanced transcription activity (Fig. 7B). Although about 2-fold increases in reporter gene activity are rather moderate, the data suggest that SNPs in the promoter region of Mcp-2 contribute to the increased expression of Mcp-2.

By analyzing DNA sequences in the putative promoter regions of the Mcp-2 and Mcp-4 genes, we found an extra CANNTG motif or E-box in both gene variants from B6-cma mice (see Fig. 2B). The E-box provides binding site for transcription factors including the microphthalmia-associated transcription factor (Mitf), a member of the basic helix-loop-helix leucine zipper protein family known to be involved in the regulation of mouse chymases [4]. To examine if this additional E-box facilitates Mitf transcription activity, we cloned the A isoform of Mitf from BMMCs of B6-cma mice into the pcDNA3 vector, and the cDNA insert was verified by sequencing. The Mitf-A construct was used to transfect NIH3T3 cells together with the Mcp-2P or Mcp-2Pv reporter gene constructs, and the plain pcDNA vector was used as control. Data in Fig. 7C demonstrates that co-expression of Mitf-A caused over 3-fold increases in reporter gene activity (p<0.001). However, the Mcp-2P and Mcp-2Pv reporter constructs displayed nearly equal transcription activities with or without the expression of Mitf-A (p>0.2). Therefore, the extra CANNTG motif in the promoter region of the variant Mcp-2 gene does not contribute to the increased gene expression in response to Mitf.

## Discussion

By serendipity, we obtained B6-cma, a congenic strain of mice, with high expression of chymases Mcp-2 and Mcp-4 in mast cells. The overexpression is associated with gene variants originated from DBA/2 mice that were also found to express the chymases at the same level. We thus established useful mouse models to study the function of chymases and their implications in human diseases. B6-cma mice are superficially normal. Detailed phenotypic characterization of these mice in comparison with wild type C57BL/6 mice is under way.

Comparative studies of different strains of mice have provided important information about associations of specific genes with phenotypes. DBA/2 mice are more susceptible to the development of atherosclerosis [15]–[17]. However, the genes involved are not clear. We believe that overexpression of Mcp-2 and Mcp-4 chymases may play an important role in this process. Chymases are known as leucocyte chemoattractants and have been shown to induce apoptosis of vascular smooth muscle cells, endothelial cells, and macrophages [18]–[21], which all could contribute to plaque formation and stability. They can also convert angiotensin I into the proinflammatory, vasoactive angiotensin II [22]. Importantly, Mcp-4-positive mast cells are accumulated in atherosclerotic lesions, and they promote atherosclerosis by releasing pro-inflammatory cytokines [23]. Therefore, chymases are targets for cardiovascular diseases [24]. In fact, chymase inhibition reduces atherosclerotic plaque progression and improves plaque stability in ApoE−/− mice [25]. Our congenic B6-cma mice thus provide an excellent system to study the involvement of chymase in atherosclerosis.

DBA/2 mice are also more susceptible to the development of autoimmune myocarditis [26], [27]. This may also be related to increased activity of chymases. Murine mast cell chymases have been shown to be involved in many pathological events related to immune responses. An earlier study demonstrated a much reduced rate of autoimmune arthritis in Mcp-4 knockout mice induced by collagen and anti-collagen antibodies [28]. Mcp-2 and Mcp-4 have also been shown to play a protective role in the sepsis model induced by cecal ligation and puncture [6], [29], and Mcp-4 has also been shown to protect the host from extensive allergic airway inflammation [30], [31].

In consistence with the pathological role of chymase in murine models, studies have demonstrated strong associations of chymse with human diseases. Two major SNPs, one located in the promoter region and another in intron 2, have been identified. They have found to be associated with atopic skin disorders [32]–[34] and cardiovascular disease [35]–[39]. The mechanism underlying the association is not known. Our study indicates that gene variations or SNPs affect gene expression of chymases in mice. It is not known if these SNPs affect expression of the enzyme in human mast cells.

In the human genome, there is only one mast cell chymase which is encoded by the *CMA1* gene, whereas mouse has 6 chymases including Mcp-1, 2, 4, 5, 9, and 10. Based on sequence similarity, Mcp-5 is the closest homolog to human chymase. However, Mcp-5 has elastase- rather than chymotrypsin-like substrate specificity [40]. Mcp-4 is the mouse chymase that has substrate specificity highly similar to that of human chymase [41], [42]. Mcp-2, on the other hand, has been shown to lack enzymatic activity [43]. However, controversial results came from studies by Orinska et al demonstrating that deletion of IL-15 increases chymase activities through specific upregulation of Mcp-2 expression, but not the other chymases [6]. Therefore, further studies are needed to clarify this, and our study provided an excellent system for this.

Overexpression of Mcp-2 and Mcp-4 may be caused by altered transcription. Regulation of chymases at the transcriptional level has been extensively studied. A major transcription factor involved is Mitf that contains both basic helix-loop-helix and leucine zipper structural features. Binding of Mitf to promoter element CANNTG significantly enhanced the expression of mMCP-2, -4, -5, -6, and -9 genes in C57BL/6 mice [4]. Interestingly, our study revealed that SNPs cause generation of an extra CANNTG site in both Mcp-2 and Mcp-4 genes of the DBA/2 origin. However, reporter gene assays did not show any increased transcriptional activity due to this additional binding site (Fig. 7C). Bifunctional transcription factors C/EBPβ and YY1 have also been implicated in the regulation of Mcp-2 gene expression and are thought to be responsible for the negative transcriptional regulation of Mcp-2 through intracellularly retained IL-15 [5], [6]. C/EBPβ is considered as a negative regulator of Mcp-2 expression. We searched consensus sequences for C/EBPβ and YY1 binding sites within the putative promoter regions of Mcp-2 and Mcp-4 (see Fig. 2B). There are multiple putative C/EBPβ and 4 YY1 binding sites in these regions. SNPs in the region did not change any of these sites in the Mcp-2 promoter but illuminated one consensus C/EBPβ site from Mcp-4. Therefore, our analysis did not support a strong correlation of Mcp-2 expression with the C/EBPβ binding. However, whether or not it contributes to the expression of Mcp-4 needs further investigation.

Expression of chymases in mast cells is controlled at the post-transcriptional and epigenetic levels. An earlier study demonstrated that the high steady-state level of the Mcp-5 transcript over those of Mcp-1, 2, and 4 is due to rapid turnover of transcribed mRNAs of the latter [7]. The transcripts of Mcp-2 and Mcp-4 but not Mcp-5 contain multiple UGXCCCC motifs in their 3′-UTRs, and these potential *cis*-acting elements are thought to be responsible for the reduced stability of the Mcp-2 and Mcp-4 transcript [4], [7]. However, analyses of coding DNA of mouse chymases in the GenBank database revealed that the 3′-UTRs of Mcp-2 and Mcp-4 transcripts from DBA/2 mice has three and six such motifs, respectively, in comparison with three from each gene in C57BL/6 strain. Therefore, the difference in the numbers of UGXCCCC motifs does not explain the overexpression of Mcp-2 and Mcp-4 in mast cells of B6-cma and DBA/2 mice. Regulation at the epigenetic level may also contribute to the overexpression of the Mcp-2 and Mcp-4 genes of the DBA/2 origin. In fact, an earlier study demonstrated hyper-transcription of the mMCP-2 gene in IL-15-deficient BMMCs is associated with histone acetylation and, intriguingly, with methylation of non-CpG dinucleotides within the MCP-2 promoter [5]. It will be interesting to investigate if epigenitic regulation of chymase expression is altered in B6-cma and DBA/2 mice.

Above all, the overexpression of Mcp-2 and Mcp-4 in mast cells from B6-cma and DBA/2 mice is unusually high, represented by over 1000-fold increases in mRNA transcripts and over 20% contributions of the expressed Mcp-2 and Mcp-4 proteins to total protein contents in cells. The mechanism underlying such a high level of overexpression warrants further investigation. Genes encoding for the 7 mouse chymases are clustered on chromosome 14 with Mcp-2 and Mcp-4 located in the middle in a tail-to-tail orientation. It is not known whether additional elements outside of the chymase cluster locus contribute to overexpression of Mcp-2 and Mcp-4. Further crossing of our B6-cma mice with C57BL/6 mice followed by genotyping of the Mcp-2 and Mcp-4 gene variants may help to answer the question. It will also be interesting to see if the Mcp-2 and Mcp-4 gene variants can be segregated through breeding. Finally, the ongoing mouse genome project will provide invaluable data for us to dissect the differential regulation of chmyases in different strains of mice.
